# Effects of Different Drying Methods and Storage Time on Free Radical Scavenging Activity and Total Phenolic Content of *Cosmos caudatus*

**DOI:** 10.3390/antiox3020358

**Published:** 2014-05-07

**Authors:** Ahmed Mediani, Faridah Abas, Chin Ping Tan, Alfi Khatib

**Affiliations:** 1 Department of Food Science, Faculty of Food Science and Technology, Universiti Putra Malaysia, 43400 Serdang, Malaysia; E-Mail: medianiahmed47@gmail.com; 2 Laboratory of Natural Products, Institute of Bioscience, Universiti Putra Malaysia, 43400 Serdang, Malaysia; E-Mail: alfikhatib1971@gmail.com; 3 Department of Food Technology, Faculty of Food Science and Technology, Universiti Putra Malaysia, 43400 Selangor, Malaysia; E-Mail: tancp@putra.upm.edu.my; 4 Faculty of Pharmacy, International Islamic University Malaysia, 25200 Kuantan, Malaysia

**Keywords:** *Cosmos caudatus*, free radical scavenging activity, total phenolic content, drying, storage

## Abstract

The present study was conducted to determine the effect of air (AD), oven (OD) and freeze drying (FD) on the free radical scavenging activity and total phenolic content (TPC) of *Cosmos caudatus* and the effect of storage time by the comparison with a fresh sample (FS). Among the three drying methods that were used, AD resulted in the highest free radical scavenging activity against 1,1-diphenyl-2-picrylhydrazyl (DPPH) (IC_50_ = 0.0223 mg/mL) and total phenolic content (27.4 g GAE/100 g), whereas OD produced the lowest scavenging activity and TPC value. After three months of storage, the dried samples showed a high and consistent free radical scavenging activity when compared to stored fresh material. The drying methods could preserve the quality of *C. caudatus* during storage and the stability of its bioactive components can be maintained.

## 1. Introduction

*Cosmos caudatus* is a traditional vegetable locally known as “ulam raja” in Malaysia and is named “kenikir” in Indonesia, “tagalog” in the Philippines and “daoruang-phama” in Thailand. The plant originated from tropical Central America and belongs to the Asteraceae family [1]. It is a large annual plant, approximately 1.5 m tall, with pleasant, colorful flowers and beautifully shaped leaves. It is considered an herb and vegetable of great importance in traditional medicine and nutrition among the populations of tropical countries, especially in Malaysia. “Ulam raja” grows quickly and easily in fertile soil, and it does not need much attention during the growing period; thus, it is of great prominence to the people of tropical regions for its nutritional, medicinal and economical values.

In Malaysia, people consume the plant fresh in salads or cooked, and its aroma and taste enhance the appetite. “Ulam raja” has been used traditionally to support the rigidity of bones, for blood circulation and to treat high blood pressure [1]. Furthermore, it is considered to be a vital component of the daily diet.

The use of medicinal herbs as a source of antioxidants is significant because they are often utilized to heal chronic maladies [2,3,4]. Moreover, the antioxidants in herbs have been confirmed to combat cancer, diabetes and cardiovascular diseases [5]. The defensive effects against these maladies are probably provided by the incidence of numerous functional metabolites, such as phenolic compounds, vitamin C, vitamin E, provitamins and minerals [6,7,8]. Antioxidant compounds in herbs include both dietary ones, such as vitamins A, C and E, and also reasonable quantities of beta-carotene, carotenoids and total phenolic compounds which are considered non-nutritional antioxidants. Free radical scavenging activity is an antioxidant mechanism that is provided by the contribution of several metabolites extracted from plants [9,10,11,12].

Medicinal herbs are usually subjected to drying and longtime storage during production, and drying is considered a beneficial way to protect their phytochemical efficiency. Furthermore, it also has the advantage of reducing the cost of final product, as transportation and storage costs are determined by product weight [5]. Although the antioxidant activity of *C. caudatus* has been reported previously [1], no study has ever been conducted on the effect of the processing and storage time on the antioxidant activity. Therefore, the aim of this work was to evaluate the effect of drying methods and storage time on the free radical scavenging activity and total phenolic content of *C. caudatus*. The study is of great importance to generate information about the drying process in preserving food quality and bioactivity by avoiding the degradation of metabolites.

## 2. Experimental Section

### 2.1. Chemicals

Folin-Ciocalteau phenol reagent, gallic acid, sodium carbonate (Na_2_CO_3_), 1,1-diphenyl-2-picrylhydrazyl (DPPH), methanol was supplied by Merck (Darmstadt, Germany). Water was purified by a Milli-Q system (Millipore, Bedford, MA, USA).

### 2.2. Plant Material

Seeds were provided by the Institute of Bioscience, Universiti Putra Malaysia (UPM) and were planted in the UPM Agricultural Park. A plot with an area of 10 m^2^ was established in an open field with a temperature of 30 °C during the day and 21 °C during the night; the relative humidity ranged from 80% to 90%. The plants were exposed to 12 h per day of sunlight. The plot was surrounded by a wall. The soil was treated, turned and covered with a black plastic. The seeds were planted in equidistant holes that were made in 2 columns in the plastic; every column contained 19 holes. During sowing, 5 seeds were placed in the center of each hole, and were later thinned to one plant per hole. The irrigation was carried out automatically with a hydraulic system and manually. Fertilization with organic fertilizer was executed according to the experience of the center workers; the fertilization was repeated every two weeks.

For a comparison of the three drying types; air drying (AD), freeze drying (FD) and oven drying (OD), eight-week-old plants were harvested randomly at the same time in the early morning to ensure the consistency of metabolite contents. The leaves were cut with laboratory scissors without taking any damaged leaves. Each sample was put in one plastic bag separately, which corresponded to one replication. After harvesting, the samples were washed with distilled water and dried with tissue paper. One batch of 600 g of fresh sample was subjected to freezing at −20 °C for three months to study the effect of storage. Similarly to fresh samples, the dried ones were also vacuum-packed and kept at −20 °C for evaluating the effect of storage after drying after three months.

### 2.3. Drying Process

The samples were labeled and subjected immediately to drying by one of three methods (air, freeze and oven). Fresh leaves (200 g) were used for each drying method with six replicates to record the dry weight. The same procedures were performed for all of the samples. OD depends on the optimized conditions of the drying time and oven temperature, which were optimized previously [13], at 44.5 °C for 4 h in a Mammert laboratory oven under forced-air ventilation. Freeze-dried raw material was prepared by subjecting it to freezing at −20 °C in freezer for 1 day and then lyophilization overnight in a freeze drier until the weight remained constant. Air-dried samples were exposed to ambient temperature in the laboratory for 6 days at 25 °C. A portion of the three dried samples were kept in aluminum containers and subjected to freezing at −20 °C for three months to evaluate the effect of storage on their antioxidant activity (AA) and TPC. Moisture content was determined from sample weight loss after drying using air-oven at 110 °C for 4 h following AOAC method 920.87 [14].

### 2.4. Extraction of Antioxidant Compounds

A portion of each dried sample (4 g) was ground into fine powder and immersed in 100 mL of 80% methanol in a 250 mL amber bottle. For the fresh samples, 20 g of crunched leaves were also immersed with the same amount of solvent. The bottles were shaken to mix the powder with the solvent, and the mixtures were subjected to vibration in a sonicator bath for 1 h at ambient temperature (25 °C). This process time was divided into two intervals, with a break of 15 min to avoid a temperature increase of the bath and mixtures. The mixtures were then left to cool at room temperature, and they were filtered with filter paper and cotton twice to remove any debris. Next, the solvents were evaporated by rotary evaporation under a partial vacuum at 40 °C. The concentrated extracts were stored in amber bottles in a freezer for future utilization.

### 2.5. Free Radical Scavenging Assay

A modified method was used for performing the DPPH assay [5]. An aliquot (1 mL) of each diluted sample (6.25, 12.5, 25, 50, 100 and 200 μg/mL) and the solvent as a control were mixed with 2 mL of DPPH solution (5.9 mg/100 mL of the solvent) in a test tube. The tubes were agitated to homogenize the mixtures and incubated in the dark for 30 min before the recording of their absorbance at a wavelength of 517 nm. The results are expressed as the IC_50_. For the accuracy of the results, six replication of each sample was used. The radical scavenging activity was measured as a decrease in the absorbance of DPPH. A lower absorbance indicates a higher free radical scavenging activity.

### 2.6. Total Phenolic Content Assay

This assay was performed according to Chan *et al.* [5]. Gallic acid was used as a standard in evaluating the TPC. An aliquot (300 μL) of 200 μg/mL of each sample (in six replicates) was transferred to a test tube and mixed with 1.5 mL of diluted Folin-Ciocalteau reagent, which was diluted ten times. After incubating for 10 min, 1.2 mL of sodium carbonate (7.5 g/100 mL) was added. The mixtures were homogenized by shaking the tubes with a vortex mixer. The tubes were then incubated in the dark for 30 min before the absorbance was determined at 765 nm. The results are reported in mg gallic acid per 100 g of sample. The concentration of gallic acid is expressed in mg/mL.

### 2.7. HPLC Method

The high-performance liquid chromatography (HPLC) profiling of the three dried samples was performed according to Mustafa *et al.* [15]. A reversed-phase HPLC (Waters 2487, Waters, Milford, MA, USA) with a UV detector and equipped with integrated software was used for the analysis. The analyte separation was carried out using a Hypersil GOLD C_18_ column (5 μm, 150 mm × 4.6 mm, Waters, Milford, MA, USA) with a gradient mobile phase composed of methanol (solvent A) and water (solvent B) containing 0.1% trifluoroacetic acid. The flow rate was maintained at 0.8 mL/min for all of the samples. The gradient program was carried out as the following sequence of solvent B: 100% in 0 min, 50% in 20 min, 0% in 30 min and 100% in 40 min.

The samples and standards were prepared by dissolving 3 mg of the dried material extract in 3 mL of 80% methanol. Standards were chosen depend on the previous literature on *C. caudatus*. Quercetin rhamnoside and glucoside were previously isolated from this plant by our group. The injected volumes of the samples were filtered by a 0.2 μm nylon membrane fitted into a 2 mL screw-capped sample vial. The injection was performed for all of the samples with a consistent volume of 20 μL. The detection of the phenolic peaks was achieved at wavelengths of 280 and 340 nm. The retention time of each sample peak was compared to the one of standards and the peak intensity was checked by overlaying the chromatograms.

### 2.8. Statistical Analysis

The results are reported as the mean of six measurements and the standard deviation. One-way ANOVA with Tukey comparison test was used in evaluating the significant difference between the samples with a confidence interval of 95%. The correlation between the DPPH and TPC was also performed by a Pearson test. All the analyses were performed in Minitab 14 software (Minitab Inc., State College, PA, USA).

## 3. Results and Discussion

We noted a distinct, pleasant aroma and green color of the *C. caudatus* sample when the sample was processed, which was maintained between each drying method, although the air-dried sample displayed the features to the highest degree.

The onset of plant metabolite degradation potentially begins at harvest, and the continuing activity of enzymes would contribute to the variation in antioxidant power of a plant after that. However, the exposure to some processing methods may contribute to this decline in plant quality. This variation among three different types of drying methods (AD, FD and OD) was studied in addition to the effect of drying on samples stored for three months by a comparison with FS.

### 3.1. Variation of Dry Weight and Moisture Content among the Dried Samples

Due to the retardation of enzymatic reactions, drying is usually applied in the processing of herbs to preserve their bioactivity and to expand the shelf life [16]. The dry weights of the differently dried samples are shown in Table 1. Although there was no significant variation (*p* > 0.05) among their weights, the OD resulted in the highest decrease. The same trend was marked for their moisture contents (Table 1). The productivity of these drying techniques was quite equivalent in terms of the obtained drying materials and their moisture contents. Hence, the hypothesis of moisture impact contribution on AA and TPC variations of dried samples is excluded. However, moisture loss could affect organoleptic parameters, such as color and taste.

**Table 1:** Dry weight and moisture content of samples processed by three drying methods.

Samples	Dry Weight (g)	Moisture Content (%)
Air dried	21.2 ^a^ ± 0.4	8.8 ^a^
Freeze dried	21.09 ^a^ ± 0.6	8.5 ^a^
Oven dried	20.77 ^a^ ± 0.3	9.4 ^a^

Values are the means ± standard deviation of six replicates. ^a^ The same small letters within different columns statistically not significant (*p* > 0.05) difference.

The drying methods produced materials that was amenable to grinding and increased the extraction efficiency. The results also indicated a change in color after drying [17]. All of the processed samples exhibited a pale green color; however, the oven-dried sample showed a darker color than the others. The OD temperature potentially leads to the reduction in quality that is associated with changes in color because of the caramelization of the Maillard reaction, enzymatic degradation and pigment loss [18].

### 3.2. Drying Influence on Phenolic Compounds

**Figure 1:**
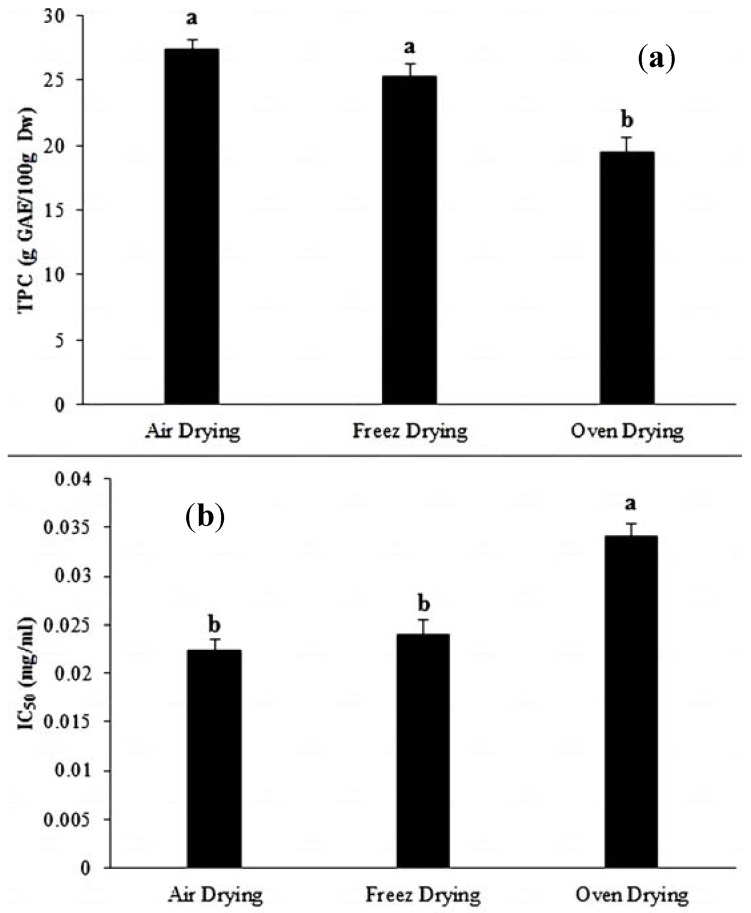
(**a**) Influence of the three drying methods on total phenolic content (TPC) of *C. caudatus*; (**b**) Influence of the three drying methods on the free radical scavenging activity (IC_50_) of *C. caudatus*. ^a,b^ Each different small letters above the columns indicates a statistically significant difference (*p* < 0.05; *n* = 6).

Generally, drying treatment is to preserve the food samples by removing the water. Some phytochemicals degradation can occur if the drying conditions are not appropriate. The relative instability of most phenolic compounds from plants may indicate a sensitivity of these compounds to drying treatments [3]. However, under some circumstances, drying could be beneficial in protecting these compounds and may not reduce their quality. For instance, natural antioxidants, such as lycopene, were reported to possess a latent stability to drying processes [19,20]. Manufacturing processing, such as peeling and storage, could also impact the variation of natural antioxidants by enhancing enzymatic reactions. Previous studies have indicated that the temperature of drying leads to a notable decline in phytochemicals [21]. As determined by the Folin-Ciocalteau method, which measures the capability of the TPC to reduce the Folin reagent [22], the total phenolic contents are shown in Figure 1a. In our study, we found that FD was preferable in comparison to oven drying, which was found to induce the degradation of bioactive compounds in plants by the effects of the heat treatment. This finding is in agreement with several research reports [5]. Moreover, we found that AD resulted in the highest value of TPC, followed by freeze drying and, lastly, oven drying.

However, the difference in TPC was not significant at *p* > 0.05 among the drying methods (except for the OD) with values of 27.4, 25.3 and 19.44 for AD, FD and OD, respectively.

AD produced the highest values for the TPC. The explanation of this result might be due to the slow loss of moisture without any pressure at ambient temperature for one week. This slow action might associate with the liberation of phenolic compounds under stress from herbs, which are performed it as defending system.

The OD showed the lowest value for the TPC, at 19.5 g GAE/100 g, which could be explained by the influence of the heat treatment that might cause a degradation of these compounds. Enzymatic and thermal degradation was possibly caused by the decline in TPC after drying. It has been reported that the activity of polyphenol oxidase after thermal processing caused the decline of the TPC before its total inactivation [3,23]. However, this finding of the current study does not support some previous research. Thus, the influence of drying on the TPC is dependent on the plant species and the stability of the cell wall [21].

### 3.3. Drying Impact on DPPH Radical Scavenging Activity

Almost all chronic diseases are associated with the danger of free radicals, which are also considered a cause of advanced aging [15]. Moreover, the obligation to replace marketed synthetic antioxidants that cause additional problems is one motivation in extracting safe natural antioxidants. Therefore, elucidating alternative solutions from herbs is becoming an important task among researchers, and the evaluation of the bioactivity of these herbs essential for this task. Many methods have been applied to evaluate the antioxidant activity of plant material, and the variation between these analyses can often be attributed to different mechanism involved. However, almost all of these methods are based on the estimation of the inhibition of oxidizing agents by a sample, where the interaction between the sample and oxidizing agents provides the antioxidant activity. The radical scavenging assay is the most widely used analysis in evaluating this activity in phytochemistry. It is a simple and rapid test that is based on the reaction rate between a stable free radical, 1,1-diphenyl-2-pycrylhydrazyl (DPPH) and antioxidants. The availability of DPPH radicals commercially is another reason for its broad utilization [24].

When a hydrogen atom or electron was transferred to the odd electron in DPPH, resulted in the change in the color of DPPH from purple to yellow and measured by spectrophotometry at a wavelength of 517 nm. The results are often expressed as IC_50_, which is the half the equivalent inhibitory concentration to give a 50% effect in scavenging the free radicals. Therefore, a lower IC_50_ value indicates a high radical scavenging ability.

The data present in Figure 1b showed that *C. caudatus* possessed a high AA. However, there was variation among the drying methods, which caused a reduction in this activity. The OD resulted in the lowest value of free radical scavenging inhibition, whereas there was no significant difference *p* > 0.05 between the FD and AD. However, AD exhibited the highest AA. There was an insignificant difference between the IC_50_ of the freeze- and air-dried samples, with values of 0.0223 and 0.023 mg/mL, respectively. The OD showed a significant decrease compared to the other two methods, with a value of 0.034 mg/mL.

The explanation of this result could be due to the impact of the heat treatment, which caused enzymatic denaturation by the Maillard reaction [18]. Additionally, the degradation of bioactive compound could also be a cause of this variation.

### 3.4. Effect of Storage

Recently, the rate of chronic diseases has increased dramatically. The discovery of natural antioxidants that combat theses maladies is the concern of researchers. The preservation of the safety and quality of herbs is another aspect that should also be taken into consideration.

Today, consumers are informed about the influence of processing operations on food quality and realize the danger of synthetic antioxidants in preservation. Therefore, the mission of researchers is to convince them about the efficiency of these methods in extracting and conserving the benefits of natural antioxidant sources. Extra research is required to overcome the outlook of drying benefit in food preservation during storage.

Storage is applied in herb manufacturing before processing for several purposes. This operation requires some conditions which could be offered by drying the raw materials. The ease of handling and estimating activities of materials is also an important advantage of drying during storage. The association of drying with freezing during storage could augment the shelf life of the product.

**Figure 2:**
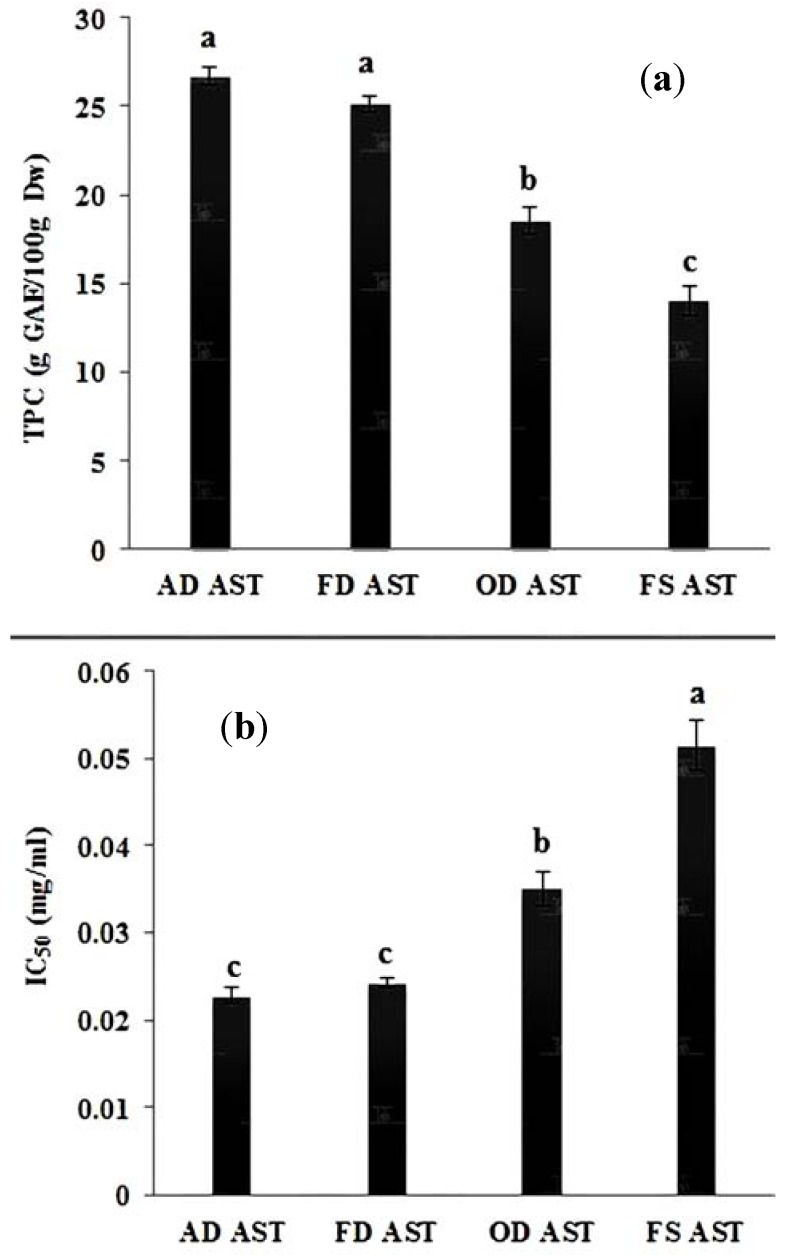
(**a**) Evaluation of the TPC variation after three months of storage of processed *C. caudatus* samples; (**b**) Evaluation of the IC_50_ difference after three months of storage of processed *C. caudatus* samples. AD AST air-dried sample after storage time, FD AST freeze-dried sample after storage time, OD AST oven-dried sample after storage time and FS AST fresh sample after storage time. ^a–c^ Each different small letters above columns indicates a statistically significant (*p* < 0.05; *n* = 6) variation.

All of the dried samples (AD, FD and OD) exhibited higher AA and TPC values than the fresh sample after the storage time (FS AST). The AA values for AD, FD and OD (0.023, 0.024, and 0.035 mg/mL, respectively) were higher than that of FS AST (0.056 mg/mL). We found no significant differences (*p* > 0.05) among the dried materials before and after storage; they were found to exhibit a higher antioxidant activity and TPC value than the fresh sample after storage (FS AST). A possible explanation for this might be that the proper storage at −20 °C of dried materials directly after drying helped to halt the degradation of compounds. The low temperature was not an appropriate condition for enzymatic reactions that cause a decrease in the antioxidant capacity of herbs. The removal of the moisture content by drying is another reason that facilitates the preservation of the bioactivity of the dried materials during storage. It has been found that the bioactivity of fresh herbs exhibited an inferior value than dried herbs. However, the incapability of fresh herbs to maintain the same activity during storage is the reason why they are processed. The severe chilling injury might be the cause of oxidation stress and the decline of the TPC among fresh herbs because of the high moisture content (Figure 2a,b). The cause of chilling injury might be due to the interaction between the phenols and polyphenol oxidase, which might also affect the TPC value of *C. caudatus*. The fragility of the cell wall, which might be caused by drying operations, is another explanation of the similarity of the antioxidant capacity and the TPC of the dried samples after storage and may provide great extraction efficiency due to the easy liberation of metabolites responsible for these bioactivities. In contrast, after storage, the FS AST might lose antioxidative metabolites when it comes in contact with ambient air due to the activity of enzymes. This mechanism of degradation was not found for the dried samples, which had a low water activity. Therefore, the enzyme does not have the appropriate conditions for activity.

The storage analysis would favor the use of any of the three drying methods over the stored fresh herbs because their AA and TPC were found to be higher than the fresh herbs. The alteration of the quality of the fresh herbs was significant compared to the dried samples.

**Figure 3:**
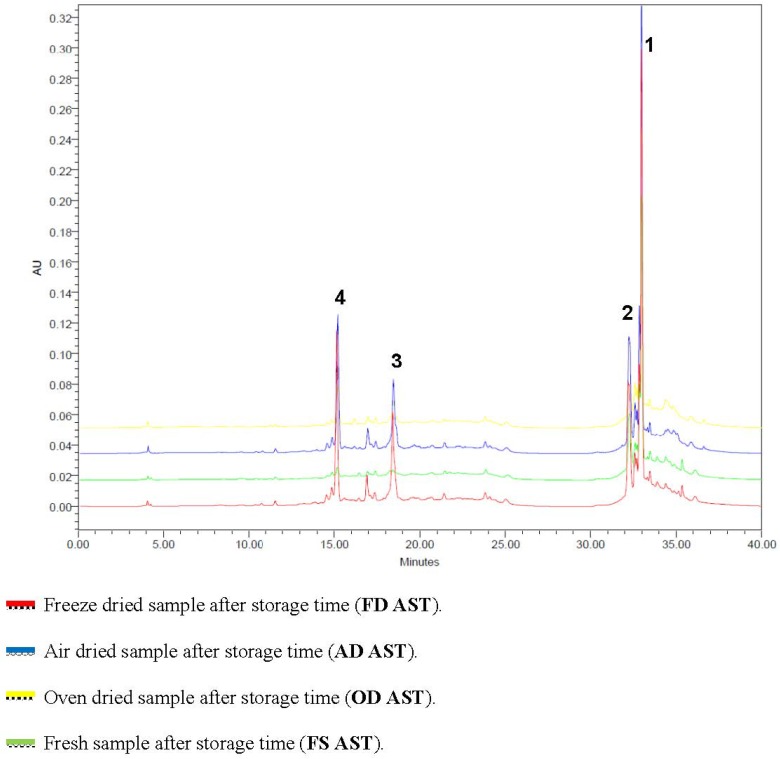
Overlaid chromatograms of *C. caudatus* samples stored for three months with different drying methods. AD AST: air-dried sample after storage time, FD AST: freeze-dried sample after storage time, OD AST: oven-dried sample after storage time and FS AST: fresh sample after storage time. Identified peaks: 1, quercetin rhamnoside; 2, quercetin glucoside; 3, rutin; 4, chlorogenic acid.

Results from the overlaid HPLC chromatograms (Figure 3) confirmed the results of the highest antioxidant ability and TPC values for the dried *C. caudatus versus* the stored fresh material. Variations in the peak intensity of the identified compounds (quercetin rhamnoside and glycoside, rutin and chlorogenic acid) might be due to the difference in the results for the TPC and AA. All of the metabolite peaks in dried materials after storage were higher than those of the fresh samples in term of the quantities. By comparing the drying methods, the AD and FD methods showed a non-significant difference in the quantity of the compounds, which were higher than the OD method.

### 3.5. Correlation between DPPH and TPC

There was a strong correlation between the antioxidant activity and TPC, with *R*^2^ = 0.95 for all of the analyses. The potential contribution of the phenolic compounds to the antioxidant capacity of *C. caudatus* is the explanation for this finding.

This finding corroborates the previous results that reported a strong relationship between them [10,15]. However, there are some studies that reported a weak relationship between the total phenolic compounds and AA in plant extracts. For instance, a lack of correlation between both assays in edible plants from Finland and wheat was reported [25,26].

## 4. Conclusions

This study has explained the central importance of drying methods in preserving the phytochemical compounds of *C. caudatus*. The results indicate the variation of the AA and TPC among the methods of AD, FD and OD. However, the bioactivity of the samples was higher than the fresh sample after storage. In this study, it seems that AD has the minimum influence on the phytochemical compounds of herbs, and this method can be applicable due to the acceptable levels of AA and TPC and its low cost. The necessity of drying prior to storage is to maintain the quality of the herbs or, at least, to reduce the negative impact. The results from this study will be used as a guide to incorporating *C. caudatus* as an ingredient in other food products.
